# The primary ciliary dyskinesia-related genetic risk score is associated with susceptibility to adult-onset asthma

**DOI:** 10.1371/journal.pone.0300000

**Published:** 2024-03-08

**Authors:** Rie Shigemasa, Hironori Masuko, Hisayuki Oshima, Kentaro Hyodo, Haruna Kitazawa, Jun Kanazawa, Yohei Yatagai, Hiroaki Iijima, Takashi Naito, Takefumi Saito, Satoshi Konno, Tomomitsu Hirota, Mayumi Tamari, Tohru Sakamoto, Nobuyuki Hizawa

**Affiliations:** 1 Department of Pulmonary Medicine, Faculty of Medicine, University of Tsukuba, Tsukuba, Japan; 2 Tsukuba Medical Center, Tsukuba, Japan; 3 National Hospital Organization Ibaraki Higashi National Hospital, Tokai, Japan; 4 Department of Respiratory Medicine, Faculty of Medicine, Hokkaido University, Sapporo, Japan; 5 Research Center for Medical Science, The Jikei University School of Medicine, Tokyo, Japan; Kaohsiung Medical University Hospital: Kaohsiung Medical University Chung Ho Memorial Hospital, TAIWAN

## Abstract

**Background:**

Disturbance of mucociliary clearance is an important factor in the pathogenesis of asthma. We hypothesized that common variants in genes responsible for ciliary function may contribute to the development of asthma with certain phenotypes.

**Methods:**

Three independent adult Japanese populations (including a total of 1,158 patients with asthma and 2,203 non-asthmatic healthy participants) were studied. First, based on the ClinVar database (https://www.ncbi.nlm.nih.gov/clinvar/), we selected 12 common single-nucleotide polymorphisms (SNPs) with molecular consequences (missense, nonsense, and 3’-untranslated region mutation) in 5 primary ciliary dyskinesia (PCD)-related genes and calculated a PCD-genetic risk score (GRS) as a cumulative effect of these PCD-related genes. Second, we performed a two-step cluster analysis using 3 variables, including PCD-GRS, forced expiratory volume in 1 second (%predicted FEV_1_), and age of asthma onset.

**Results:**

Compared to adult asthma clusters with an average PCD-GRS, clusters with high and low PCD-GRS had similar overall characteristics: adult-onset, female predominance, preserved lung function, and fewer features of type 2 immunity as determined by IgE reactivity and blood eosinophil counts. The allele frequency of rs1530496, a SNP representing an expression quantitative trait locus (eQTL) of *DNAH5* in the lung, showed the largest statistically significant difference between the PCD-GRS-High and PCD-GRS-Low asthma clusters (p = 1.4 x 10^−15^).

**Conclusion:**

Genes associated with PCD, particularly the common SNPs associated with abnormal expression of *DNAH5*, may have a certain influence on the development of adult-onset asthma, perhaps through impaired mucociliary clearance.

## Introduction

Asthma is a chronic obstructive airway disease with multiple phenotypes that may differ in disease pathobiology; the disease is caused by complex gene-environmental interactions. Several genome-wide association studies (GWASs) of asthma have been conducted to date [1, 2], and some GWASs have identified novel candidate genes by focusing on specific phenotypes of asthma [3, 4]. By focusing on the adult-onset phenotype, we previously reported that *HCG22*, a member of a gene cluster encoding mucin-like proteins that is located on chromosome 6p21.3 and is also known as a candidate gene for diffuse panbronchiolitis (DPB), is associated with asthma [5]. DPB is a chronic obstructive airway disease characterized by neutrophilic bronchiolitis and mucus hypersecretion leading to impaired mucociliary clearance.

Importantly, impaired mucociliary clearance is broadly implicated in asthma pathogenesis, with or without a type 2 phenotype, and dysfunction of cilia may be in part responsible for poor mucus and/or allergen clearance from the airways [6]. Impaired mucociliary function caused by allergic immune response also weakens the barrier integrity and self-cleaning abilities of the airway epithelium making it more vulnerable to penetration of allergens as well as of infection by bacteria and viruses [7]. In a previous candidate gene analysis, KIF3A, a member of the kinesin superfamily of microtubule-associated motors that are important in the transport of protein complexes within cilia, was identified as a novel gene for childhood asthma [8].

Primary ciliary dyskinesia (PCD) is another airway disease characterized by impaired mucociliary clearance and airway neutrophilic inflammation caused by structural and/or functional abnormalities of cilia. A mutation in any one of the proteins required to build or regulate cilia can cause this disease; more than 40 related genes have been reported to date [9–11]. A previous study analyzing bronchial airway epithelial cell gene expression in relation to fractional exhaled nitric oxide (FeNO) identified a distinct asthma phenotype mainly involving cilia-related genes [12].

In the present study, given the complexity and heterogeneity of asthma, we conducted candidate gene analysis of adult asthma by hypothesizing that common functional variants at multiple PCD-related genes, rather than a rare mutation at a single gene, contribute to the susceptibility to specific asthma phenotypes in which ciliary dysfunction could play an important role in the disease pathogenesis.

## Results

The baseline characteristics of each of the 3 cohorts are shown in S1 Table. The median age of onset for patients with asthma in the 3 cohorts was approximately 40 years. Patients with asthma in Tsukuba Cohort 2 were characterized by fewer smokers (18.1%), and patients with asthma in Hokkaido Cohort were characterized by a lower prevalence of atopy (55.3%) and a higher prevalence of eosinophilic asthma (71.4%) compared to patients with asthma in the other cohorts. There was no significant association between each of the 12 single-nucleotide polymorphisms (SNPs) and the prevalence of asthma in any population (S2 Table).

The two-step cluster analysis of patients with asthma in Tsukuba Cohort 1 identified 4 clusters, which were designated Clusters T1-A to -D in the order of decreasing PCD-genetic risk score (GRS) values. Patients with asthma in Tsukuba Cohort 2 and in Hokkaido Cohort were classified into 5 distinct clusters designated Clusters T2-A to -E and Clusters H-A to -E, respectively. The clinical characteristics of the identified asthma clusters in each cohort are summarized in Tables 1a–1c and 2.

**Table 1:** **a.** Characteristics of asthma clusters identified in Tsukuba Cohort 1. **b.** Characteristics of asthma clusters identified in Tsukuba Cohort 2. **c.** Characteristics of asthma clusters identified in the Hokkaido Cohort.

**a**						
**Tsukuba Cohort 1**	**Cluster T1-A**	**Cluster T1-B**	**Cluster T1-C**	**Cluster T1-D**	**P value** §	
N	142	120	111	102		
PCD-GRS—mean (SD)	14.15 (1.31)	12.52 (2.16)	12.33 (1.95)	10.67 (1.44)	<0.001	
Age—years, median (range)	64 (29–84)	71 (25–90)	43 (19–86)	68.5 (41–89)	<0.001	
Age of onset—years, median (range)	49.5 (19–82)	50 (10–82)	7 (1–35)	60 (31–87)	<0.001	
Female—n (%)	93 (65.5)	53 (44.2)	60 (54.1)	57 (55.9)	0.0071	
BMI—mean (SD)	24.14 (4.01)	23.26 (4.41)	23.71 (4.0)	23.89 (3.97)	0.38	
FEV_1_%predicted—mean (SD)	94.56 (15.41)	52.83 (12.38)	81.21 (17.35)	98.51 (18.79)	<0.001	
Z—score FEV_1_—mean (SD)	-0.76 (1.08)	-3.76 (1.09)	-1.48 (1.46)	-0.53 (1.16)	<0.001	
FEV_1_/FVC—mean (SD)	74.62 (10.04)	57.2 (10.85)	72.27 (12.88)	73.5 (9.25)	<0.001	
Smoking status—n (%)						
Never	82 (59.4)	53 (44.5)	64 (57.7)	46 (45.5)	<0.001	
Ex-	48 (34.8)	58 (48.7)	30 (27)	50 (49.5)		
Current	8 (5.8)	8 (6.7)	17 (15.3)	5 (5.0)		
Smoking index* –n (%)						
0	82 (59.4)	53 (44.5)	64 (57.7)	46 (45.5)	0.001	
1–200	21 (15.1)	11 (9.2)	22 (19.8)	18 (17.6)		
>200	36 (25.9)	56 (46.7)	25 (22.5)	38 (37.3)		
Atopy†—n (%)	76 (63.3)	77 (69.4)	84 (84.8)	58 (63)	0.0018	
Total serum IgE (log)—mean (SD)	2.18 (0.63)	2.19 (0.67)	2.34 (0.60)	2.21 (0.63)	0.22	
Eosinophilic asthma‡—n (%)	41 (39.0)	41 (47.7)	39 (47.6)	28 (36.8)	0.35	
**b**						
**Tsukuba Cohort 2**	**Cluster T2-A**	**Cluster T2-B**	**Cluster T2-C**	**Cluster T2-D**	**Cluster T2-E**	**P value** §
N	28	63	50	28	68	
PCD-GRS—mean (SD)	14.39 (1.17)	14.19 (1.40)	12.74 (1.58)	11.14 (1.65)	10.88 (1.38)	<0.001
Age—years, median (range)	57.5 (30–72)	62 (24–75)	36 (20–73)	33.5 (20–65)	59 (31–74)	<0.001
Age of onset—years, median (range)	45.5 (30–67)	56 (24–70)	5 (0–35)	22 (1–40)	49.5 (23–69)	<0.001
Female—n (%)	17 (60.7)	41 (65.1)	18 (36)	20 (71.4)	44 (64.7)	0.0055
BMI—mean (SD)	23.81 (4.16)	23.37 (3.42)	24.33 (4.86)	23.49 (4.88)	22.71 (3.29)	0.34
FEV_1_%predicted—mean (SD)	70.20 (11.92)	108.39 (11.56)	73.19 (13.94)	107.45 (11.07)	85.99 (14.02)	<0.001
Z—score FEV_1_—mean (SD)	-2.39 (1.28)	0.10 (1.02)	-2.29 (1.19)	0.38 (1.13)	-1.40 (1.14)	<0.001
FEV_1_/FVC—mean (SD)	71.30 (13.64)	78.35 (8.17)	71.92 (13.28)	83.09 (7.83)	72.09 (9.09)	<0.001
Smoking status—n (%)						
Never	21 (75)	57 (90.5)	42 (84)	21 (75)	53 (77.9)	0.24
Ex-	2 (7.1)	3 (4.8)	2 (4)	4 (14.3)	3 (4.4)	
Current	5 (17.9)	3 (4.8)	6 (12)	3 (10.7)	12 (17.6)	
Smoking index* –n (%)						
0	21 (75)	57 (90.5)	42 (84)	21 (75)	53 (77.9)	0.33
1–200	7 (25)	6 (9.5)	8 (16)	6 (21.4)	13 (19.1)	
>200	0	0	0	1 (3.6)	2 (2.9)	
Atopy†—n (%)	20 (83.3)	35 (61.4)	44 (97.8)	17 (73.9)	36 (59)	<0.001
Total serum IgE (log)—mean (SD)	2.39 (0.54)	2.06 (0.53)	2.50 (0.52)	2.21 (0.67)	2.09 (0.70)	<0.001
Eosinophilic asthma‡—n (%)	12 (44.4)	22 (38.6)	23 (51.1)	12 (46.2)	33 (50.8)	0.67
**c**						
**Hokkaido Cohort**	**Cluster H-A**	**Cluster H-B**	**Cluster H-C**	**Cluster H-D**	**Cluster H-E**	**P value** §
N	87	89	73	85	112	
PCD-GRS—mean (SD)	14.83 (1.08)	12.78 (1.83)	12.01 (1.88)	11.46 (1.33)	11.04 (1.74)	<0.001
Age—years, median (range)	62 (21–82)	33 (16–83)	50 (18–71)	62 (36–84)	56.5 (19–80)	<0.001
Female—n (%)	46 (52.9)	44 (49.4)	44 (60.3)	50 (58.8)	79 (70.5)	0.027
Age of onset—years, median (range)	53 (19–77)	5 (0–26)	29 (0–62)	53 (33–76)	41.5 (16–75)	<0.001
FEV_1_%predicted—mean (SD)	87.50 (18.75)	85.77 (15.06)	48.91 (12.17)	69.46 (9.84)	101.89 (11.69)	<0.001
Z—score FEV_1_—mean (SD)	-1.60 (1.51)	-1.43 (1.41)	-4.75 (1.13)	-3.0 (0.74)	-0.42 (0.94)	<0.001
FEV_1_/FVC—mean (SD)	69.98 (11.71)	74.58 (10.96)	55.27 (10.87)	64.60 (10.11)	76.98 (8.64)	<0.001
Smoking status—n (%)						
Never	42 (48.3)	54 (61.4)	42 (57.5)	42 (50)	55 (49.1)	0.034
Ex-	27 (31)	16 (18.2)	18 (24.7)	26 (31)	46 (41.1)	
Current	18 (20.7)	18 (20.5)	13 (17.8)	16 (19)	11 (9.8)	
Smoking index*—n (%)						
0	42 (48.3)	54 (61.4)	42 (57.5)	42 (50)	55 (49.1)	<0.001
1–200	12 (14.1)	8 (11.6)	13 (20.3)	5 (6.4)	26 (23.9)	
>200	31 (36.5)	7 (10.1)	9 (14.1)	31 (39.7)	28 (25.7)	
Atopy†—n (%)	43 (49.4)	64 (72.7)	44 (62)	42 (50)	51 (45.9)	0.0011
Total serum IgE (log)—mean (SD)	2.27 ‘0.63)	2.55 (0.66)	2.34 (0.64)	2.22 (0.66)	2.06 (0.69)	<0.001
Eosinophilic asthma‡—n (%)	47 (58.0)	52 (60.5)	48 (73.8)	45 (57.7)	54 (49.5)	0.039

Clusters were designated A to E in order of decreasing GRS.

*Smoking index was calculated for current and past smokers by multiplying smoking dose (cigarettes per day) and duration (years smoked).

^†^Atopy was defined as a positive response (>1.0 lumicount) to at least one of the 14 inhaled allergens.

^‡^Eosinophilic asthma was defined as a peripheral blood eosinophil count of more than 300/μL or 5%.

^§^A two-tailed one-way analysis of variance (ANOVA), a Kruskal-Wallis test, or a chi-squared test was used to compare parametric continuous, nonparametric continuous, or categorical covariates, respectively. *BMI*, body mass index; *FEV*_*1*_, forced expiratory volume in 1 second; *FVC*, forced vital capacity; *PCD*, primary ciliary dyskinesia; *GRS*, genetic risk score.

**Table 2:** Summary of clinical phenotypes of the identified asthma clusters.

	Tsukuba Cohort 1	Tsukuba Cohort 2	Hokkaido Cohort
Cluster A	Female and never-smoker dominant, with preserved lung function	Late-onset, higher prevalence of atopy, with mild airflow limitation	Late-onset, heavy smoker dominant
Cluster B	Male and smoker dominant, with moderate airflow limitation	Late-onset, the lowest levels of serum IgE, with preserved lung function	Early-onset, male dominant, the highest levels of serum IgE, and the highest prevalence of atopy
Cluster C	Early-onset, the highest level of serum IgE, and the highest prevalence of atopy	Early-onset, male dominant, and the highest prevalence of atopy, with mild airflow limitation	Higher prevalence of eosinophilic asthma, with severe airflow limitation
Cluster D	Late-onset and the lowest prevalence of atopy with preserved lung function	Female dominant, with preserved lung function	Late-onset, heavy smoker dominant, with moderate airflow limitation
Cluster E	-	Late-onset and the lowest prevalence of atopy	Female dominant, preserved lung function, the lowest levels of serum IgE, and the lowest prevalence of atopy and blood eosinophilia

Clusters were designated A to E in order of decreasing GRS. *GRS*, genetic risk score.

The PCD-GRS of Cluster T1-A, Clusters T2-A and -B, and Cluster H-A were significantly higher than those of healthy participants in each population. On the other hand, the PCD-GRS of Cluster T1-D, Clusters T2-D and -E, and Clusters H-D and -E were significantly lower than those of healthy participants (Table 3).

**Table 3:** Tukey’s HSD post hoc analysis of the PCD-GRS between healthy participants and asthma clusters in each cohort.

	Tsukuba Cohort 1			Tsukuba Cohort 2			Hokkaido Cohort		
	n	PCD-GRS (SD)	P value	n	PCD-GRS (SD)	P value	n	PCD-GRS (SD)	P value
Healthy participants	565	12.71 (2.10)	-	965	12.50 (2.08)	-	673	12.46 (2.15)	-
vs. Cluster A	142	14.15 (1.31)	2.9 x 10^−13^	28	14.39 (1.17)	1.0 x 10^−05^	87	14.83 (1.08)	7.1 x 10^−13^
vs. Cluster B	120	12.52 (2.16)	0.86	63	14.19 (1.40)	1.1 x 10^−09^	89	12.78 (1.83)	0.70
vs. Cluster C	111	12.33 (1.95)	0.33	50	12.74 (1.58)	0.96	73	12.01 (1.88)	0.44
vs. Cluster D	102	10.67 (1.44)	2.2 x 10^−13^	28	11.14 (1.65)	0.0045	85	11.46 (1.33)	0.00014
vs. Cluster E	-	-	-	68	10.88 (1.38)	1.3 x 10^−09^	112	11.04 (1.74)	3.7 x 10^−11^

Clusters were designated A to E in order of decreasing PCD-GRS. *PCD-GRS*, primary ciliary dyskinesia-genetic risk score; *SD*, standard deviation.

**Fig 1:**
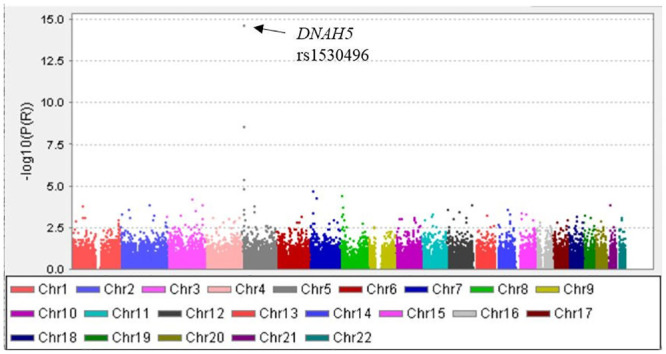
Manhattan plot of the meta-analysis of genome-wide association studies comparing asthma clusters with a high PCD-GRS (T1-A, T2-A and -B) and a low PCD-GRS (T1-D, T2-D and -E) using Tsukuba Cohorts 1 and 2 (using asthma clusters with a high PCD-GRS as a reference). The Y-axis shows the–log10 P values, and the X-axis shows the SNP chromosomal positions. The position of the DNAH5 SNP is indicated by the arrow at the top of the plot. PCD-GRS, primary ciliary dyskinesia-genetic risk score; Chr, chromosome; SNP, single nucleotide polymorphism.

To identify the genes or SNPs that contributed most strongly to the development of these particular clusters of asthma, the associations between each of 12 SNPs and asthma clusters with a higher PCD-GRS or with a lower PCD-GRS were examined in each cohort (Table 4). The SNPs, particularly those in *DNAH5*, were consistently associated with both asthma clusters with a higher GRS and a lower GRS, but the alleles associated with asthma were the opposite. When a genome-wide meta-analysis was conducted to clarify the genetic difference between asthma clusters with a higher PCD-GRS and clusters with a lower PCD-GRS using a combination of the two Tsukuba cohorts (Tsukuba Cohorts 1 and 2), the *DNAH5* gene again showed the strongest significant association with the difference in allele frequency between these two asthma groups (Fig 1). Further association analysis between asthma clusters with a higher PCD-GRS and clusters with a lower PCD-GRS in the regions within 100 kb of *DNAH5* (i.e., *DNAH5* ±100 kb), where SNP density was increased by imputation, showed that most of the significant SNPs were expression quantitative trait loci (eQTLs) of *DNAH5* in the lung (Table 5).

**Table 4:** Associations between 12 SNPs and asthma clusters with a high PCD-GRS or with a low PCD-GRS in each cohort.

*GENE*		Tsukuba Cohort 1		Tsukuba Cohort 2		Hokkaido Cohort	
	Clusters	A	D	A and B	D and E	A	D and E
	dbSNP-variant allele	OR (P value)*	OR (P value)*	OR (P value)*	OR (P value)*	OR (P value)*	OR (P value)*
*CCDC164*	rs3795958-G	1.09 (0.59)	0.71 (0.10)	1.12 (0.58)	0.76 (0.21)	0.94 (0.76)	0.81 (0.19)
*CCDC164*	rs12623642-T	0.98 (0.87)	0.75 (0.060)	1.17 (0.33)	0.87 (0.35)	1.30 (0.13)	0.80 (0.056)
*DNAH5*	rs2277046-G	1.76 (3.1x10^-5^)	0.60 (0.0013)	1.64 (0.0017)	0.80 (0.14)	1.77 (0.00043)	0.77 (0.027)
*DNAH5*	rs10513155-T	1.44 (0.026)	0.67 (0.085)	1.59 (0.015)	0.57 (0.028)	1.94 (0.00037)	0.75 (0.10)
*DNAH5*	rs1530498-G	2.30 (3.8x10^-5^)	0.48 (3.7x10^-6^)	1.92 (0.0025)	0.67 (0.013)	4.01 (1.1x10^-6^)	0.80 (0.080)
*DNAH5*	rs1530496-A	1.74 (0.00061)	0.50 (5.6x10^-6^)	2.02 (0.00024)	0.60 (0.00092)	4.11 (3.0x10^-9^)	0.68 (0.00099)
*DNAH11*	rs2285943-T	1.20 (0.18)	0.97 (0.87)	1.29 (0.10)	0.88 (0.43)	1.77 (0.00060)	0.77 (0.052)
*DNAH11*	rs10224537-G	0.99 (0.96)	0.86 (0.39)	0.79 (0.18)	0.85 (0.35)	1.58 (0.042)	0.95 (0.71)
*DNAH11*	rs2214326-A	1.36 (0.12)	0.59 (0.0033)	1.65 (0.038)	0.71 (0.058)	3.40 (0.00010)	0.90 (0.46)
*DNAH11*	rs7971-G	1.49 (0.012)	0.52 (0.0065)	1.58 (0.010)	0.74 (0.17)	1.40 (0.071)	0.78 (0.12)
*TXNDC3*	rs10250905-C	1.66 (0.00033)	0.60 (0.00085)	1.86 (0.00020)	0.52 (2.3x10^-5^)	1.44 (0.032)	0.62 (2.6x10^-5^)
*DYX1C1*	rs600753-G	1.24 (0.13)	0.65 (0.0050)	1.82 (0.0023)	0.71 (0.032)	1.81 (0.0036)	0.78 (0.043)

For each cohort, the maximum OR is graded as red, the minimum as blue, and the median as white.

*Chi-squared test was used to compare allele frequencies between adult patients with and without asthma. Clusters were designated A to E in order of decreasing PCD-GRS. *PCD-GRS*, primary ciliary dyskinesia-genetic risk score; *SNP*, single nucleotide polymorphism; *OR*, odds ratio.

**Table 5:** Meta-analysis of association studies comparing asthma clusters with a high PCD-GRS (T1-A, T2-A and -B) and a low PCD-GRS (T1-D, T2-D and -E) in Tsukuba Cohorts 1 and 2, using SNPs located in the *DNAH5* ± 100 kb region.

Chr 5	BP	dbSNP	Minor allele	MAF	Meta-P value	Meta-OR	eQTL (tissue)
	13931340	rs1530496	G	0.31	1.4 x 10^−15^	4.71	*DNAH5* (lung)
	13934349	rs12653156	C	0.40	2.4 x 10^−15^	4.33	*DNAH5* (lung)
	13933125	rs983649	C	0.29	1.7 x 10^−13^	4.19	*DNAH5*
	13954951	rs173090	T	0.27	6.3 x 10^−12^	3.59	*DNAH5* (lung)
	13939830	rs10070196	C	0.23	2.6 x 10^−9^	3.88	*DNAH5* (lung)
	13717471	rs2277046	T	0.49	7.0 x 10^−9^	2.48	-

Asthma clusters with high PCD-GRS were used as the reference. SNPs with meta-P value <10–8 are shown in the table. PCD-GRS, primary ciliary dyskinesia-genetic risk score; Chr, chromosome; BP, base pair position; SNP, single nucleotide polymorphism; MAF, minor allele frequency; OR, odds ratio; eQTL, expression Quantitative Trait Locus.

To identify the clinical characteristics of asthma clusters with high or low PCD-GRS values in greater detail, the asthma clusters with a higher PCD-GRS were combined as “PCD-GRS-Hi asthma”, and the asthma clusters with a lower PCD-GRS were combined as “PCD-GRS-Lo asthma”, and these groups were then compared to the combined remaining clusters designated as “PCD-GRS-average asthma” (Table 6). Both PCD-GRS-Hi and GRS-Lo asthma showed similar characteristics of late-onset, female-dominant, and relatively preserved lung function compared to PCD-GRS-average asthma. In addition, the prevalence of eosinophilic asthma was lowest in PCD-GRS-Hi asthma and the prevalence of atopic asthma was lowest in PCD-GRS-Lo asthma.

**Table 6:** Characteristics of combined asthma clusters with high, low, and average PCD-GRS values.

	Group1: PCD-GRS-Hi asthma	Group2: PCD-GRS-Lo asthma	Group3: PCD-GRS-average asthma	P value‡
	(Combined group of T1-A, T2-A, -B, H-A)	(Combined group of T1-D, T2-D, -E, H-D, -E)	(Combined group of the other clusters)	
N	320	395	443	
PCD-GRS—mean (SD)	14.37 (1.28)	11.02 (1.53)	12.47 (1.95)	<0.001
Female—n (%)	197 (61.6)	250 (63.3)	219 (49.4)	<0.001
Adjusted residual	1.7	2.9	-4.4	
Age—years, median (range)	61.5 (21–84)	61 (19–89)	49 (16–90)	<0.001
Age of onset—years, median (range)	51 (19–82)	51 (1–87)	20 (0–82)	<0.001
FEV_1_%predicted—mean (SD)	93.23 (18.42)	91.69 (19.05)	68.11 (20.89)	<0.001
Z—score FEV_1_—mean (SD)	-0.96 (1.42)	-1.12 (1.48)	-2.72 (1.83)	<0.001
FEV_1_/FVC—mean (SD)	74.27 (10.72)	73.30 (10.30)	65.61 (14.28)	<0.001
Smoking status—n (%)				
Never	202 (63.9)	217 (55.2)	254 (57.7)	0.10
Ex-	80 (25.3)	129 (32.8)	124 (28.2)	
Current	34 (10.8)	47 (12)	62 (14.1)	
Atopy*—n (%)	174 (60.4)	204 (55)	313 (75.6)	<0.001
Adjusted residual	-1.7	-4.7	6.1	
Total serum IgE (log)—mean (SD)	2.20 (0.61)	2.15 (0.67)	2.36 (0.64)	<0.001
Eosinophilic asthma†- n (%)	122 (45.2)	172 (48.6)	203(55.8)	0.023
Adjusted residual	-1.97	-0.85	2.62	

PCD-GRS-Hi: combined clusters with higher PCD-GRS; PCD-GRS-Lo: combined clusters with lower PCD-GRS.

*Atopy was defined as a positive response (>1.0 lumicount) to at least one of the 14 inhaled allergens.

^†^Eosinophilic asthma was defined as a peripheral blood eosinophil count of more than 300 /μL or more than 5%.

^‡^For PCD-GRS, difference was found between all groups. For age, onset age, %predicted FEV_1_, FEV_1_/FVC, and total serum IgE, difference was found between GRS-Hi group and the -average group, and between GRS-Lo group and the -average group. For categorical covariates, adjusted residuals are shown in the table. The PCD-GRS of Group 1 or Group 2 was significantly different from that of 2203 healthy individuals (p = 2.2 x 10^−6^ and p = 2.2 x 10^−6^ respectively). *FEV*_*1*_, forced expiratory volume in 1 second; *FVC*, forced vital capacity; *PCD*, primary ciliary dyskinesia; *GRS*, genetic risk score.

Because smoking has a profound effect on mucociliary function, an analysis using only nonsmokers was performed. The results did not differ from the analysis of all patients (S3 Table).

## Discussion

Stratifying asthma phenotypes using cluster analysis allowed us to successfully identify the genetic effects of PCD-related genes, especially of *DNAH5*, on asthma characterized by adult-onset, female predominance, preserved lung function, and decreased frequencies of atopic and eosinophilic asthma. Furthermore, given that both wild-type and variant alleles of *DNAH5* correlated positively with the similar phenotypes, we believe that the focus on specific asthma phenotypes by cluster analysis and the simultaneous examination of the effects of multiple SNPs using GRS helped us identify the genetic effects of the common SNPs at *DNAH5* on adult-onset asthma. In fact, previous simple GWASs of asthma have not reported any genetic association with the *DNAH5* region [13].

In a recent genetic association analysis with atopy, a significant interaction of the *DNAH5* gene was reported with *ADGRV1*, a causative gene of Usher syndrome type 2, a disease characterized by congenital deafness and retinitis pigmentosa [14]. Both genotypes of *DNAH5* (rs2134256 TT or CC) were reported to have a positive effect on atopy, depending on the genotypes of *ADGRV1* (rs17554723 AA or GG). Therefore, in the present study, the reason why both wild-type and variant alleles in *DNAH5* were positively associated with similar adult-onset asthma phenotypes may also be due to the interaction with other genes or endotypes, although significant interactions between *DNAH5* rs1530496 and the other 8 SNPs on different chromosomes examined in this study were not identified. In any case, even if the *DNAH5* gene is primarily involved in the endotypes or molecular mechanisms associated with predisposition to mucosal ciliary dysfunction and non-type 2 airway inflammation, it is clear that the genetic effects of *DNAH5* are not strong enough to cause the development of asthma with any specific phenotypes on its own. It is rather reasonable to assume that the *DNAH5* gene exerts some influence on the expression of certain asthma phenotypes as a result of its interaction with other genetic and environmental factors.

According to the GTEx portal database [15] (https://gtexportal.org/home/), the alleles that increase the expression of *DNAH5* mRNA in the lung are consistently associated with asthma with a lower PCD-GRS; the alleles that decreased the expression of *DNAH5* mRNA in the lung are consistently associated with asthma with a higher PCD-GRS. Although the functional consequences of aberrant expression of the *DNAH5* gene are not clear, a structural abnormality with an outer dynein arm (ODA) defect is the major finding in PCD, accounting for nearly 50% of cases [16]. *DNAH5* encodes the axonal heavy dynein chain of ODA and is responsible for ODA deficiency, and mutations at this gene accounted for the largest proportion (36.3%) of PCD cases in the study of Boaretto et al [17]. In the Japanese population, mutations in *DNAH5* have been found in 31% of PCD cases, second only to mutations in *CCDC164*, which have been seen in 49% of cases [18]. In PCD patients with *DNAH5* mutations, electron microscopic findings have shown two patterns of localization changes: complete loss of DNAH5 in ciliary axonemes with accumulation at the ciliary base; or DNAH5 distally absent only in the ciliary axonemes. The cilia in these patients were immotile or showed altered motility on high-speed video microscopy [19, 20].

The phenotypes with lower prevalence of atopy or with lower eosinophil counts that were identified in the present study may reflect a causal link between aberrant expression of *DNAH5* and the presence of antigen non-specific type-2 or neutrophilic airway inflammation (Table 6). When the associations of PCD-GRS with clinical information potentially related to abnormal mucociliary motility, such as exacerbations requiring antibiotics, long-term use of macrolides, and peripheral blood neutrophil counts, were examined in patients who had reliable information on these phenotypes, no clear associations were found, although the small number of eligible patients precluded any firm conclusions (S4 Table). Some patients with non-type 2 asthma, characterized by chronic productive cough and episodic infectious exacerbations, may be included in the recently proposed disease category of muco-obstructive lung disease, which includes COPD, PCD and bronchiectasis [21]. Patients in this disease category often suffer from persistent bacterial infections caused by *Haemophilus influenzae*, *Streptococcus pneumoniae*, *Moraxella catarrhalis*, *Klebsiella pneumoniae* or *Pseudomonas aeruginosa*. A complex interaction between these chronic bronchial infections and intrinsic ciliary abnormalities may underlie the etiology of this disease category. Nevertheless, given that the frequency of patients with a high or low PCD-GRS that was associated with adult-onset asthma was found to be more than 60% of all asthmatics, it is likely that the endotype associated with the PCD-GRS more broadly underlies a variety of phenotypes with or without type-2 features, rather than just defining a specific phenotype associated with PCD-like clinical features.

There are some limitations to this study. First, it is unclear how the higher or lower expression of *DNAH5* affects ciliary function. Second, the number of SNPs examined in the current study was very limited, given that we targeted only SNPs that were present either in the Infinium Asian Screening Array-24 v1.0 BeadChip [22] or the Illumina HumanHap550v3/610-Quad BeadChip [23]. Third, data were insufficient to statistically evaluate the clinical features potentially related to mucociliary dysfunction, such as the number of exacerbations requiring antibiotics, the presence of long-term macrolide use, and peripheral blood neutrophil counts. Fourth, patients with asthma COPD overlap (so-called ACOs) were not excluded. However, it is unlikely that PCD-GRS is particularly associated with COPD pathogenesis, given that asthmatics who were found to have high or low PCD-GRS levels had no apparent impaired lung function, and excluding smokers from the analysis did not significantly change the results. Finally, although *DNAH5* was significantly associated with the clusters identified in this study, the genetic backgrounds for adult-onset asthma cannot be fully explained by *DNAH5*; further validation, including the gene-gene and gene-environment interactions, will be necessary.

In conclusion, the current study suggested that impaired mucociliary clearance due to genetic susceptibility to ciliary dysfunction may underlie the pathogenesis of adult-onset asthma, which may give some insight into the complex interplay of several distinct endotypes underlying the extremely diverse clinical phenotypes of asthma patients.

## Methods

### Study population

We studied 3 independent adult Japanese populations (S1 Table). The first population (Tsukuba Cohort 1) included 565 healthy participants and 537 patients with asthma. The second population (Tsukuba Cohort 2) included 965 healthy participants and 242 patients with asthma. Patients with asthma in these 2 cohorts were recruited from the University of Tsukuba Hospital and its affiliated hospitals, and healthy participants were recruited from the health checkup center of the Tsukuba Medical Center Hospital from June 1, 2008 through March 31, 2022 [24]. The third population (Hokkaido Cohort) included 673 healthy participants and 446 patients with asthma, all of whom were recruited from the Hokkaido University Hospital [2].

In this study, asthma was defined as having recurrent episodes of 2 or more of 3 specific symptoms (coughing, wheezing, and dyspnea) known to be associated with reversible airflow obstruction and/or increased airway responsiveness [25]. The presence of eosinophilic inflammation as indicated by peripheral blood eosinophilia and exhaled NO and the presence of atopy were also used as diagnostic references. Smokers were not excluded.

The age of onset of asthma was confirmed by interview, including episodes of dyspnea, wheezing, or coughing experienced during childhood and adolescence. In cases of uncertainty, the age of the earliest respiratory symptom was considered the age of onset. Individuals with a history of asthma or chronic obstructive disease were excluded from the healthy participants.

### Ethics statement

This study was approved by the Human Genome Analysis and Epidemiology Research Ethics Committee of the University of Tsukuba and by the Human Genome/Gene Analysis Research Ethics Review Committees of the Tsukuba Medical Center, RIKEN, and the Hokkaido University School of Medicine (Ethical approval number: H29-294). Written, informed consent was obtained from each participant before the study, which was performed in accordance with the principles of the Declaration of Helsinki.

### Genotyping

For all individuals, genomic DNA was extracted from whole blood samples by an automated DNA extraction system (QuickGene-610L; Fuji Film, Tokyo, Japan). Genome-wide genotyping was carried out using Infinium Asian Screening Array-24 v1.0 BeadChip (Illumina, San Diego, CA, USA) for individuals in Tsukuba Cohort 1 [22], and Illumina HumanHap550v3/610-Quad BeadChip (Illumina) for individuals in Tsukuba Cohort 2 [23]. For individuals in the Hokkaido Cohort, genotyping of the selected SNPs was performed using the TaqMan allele-specific amplification method (Applied Biosystems, Foster City, CA, USA) [26].

### Gene and SNP selection

**Fig 2:**
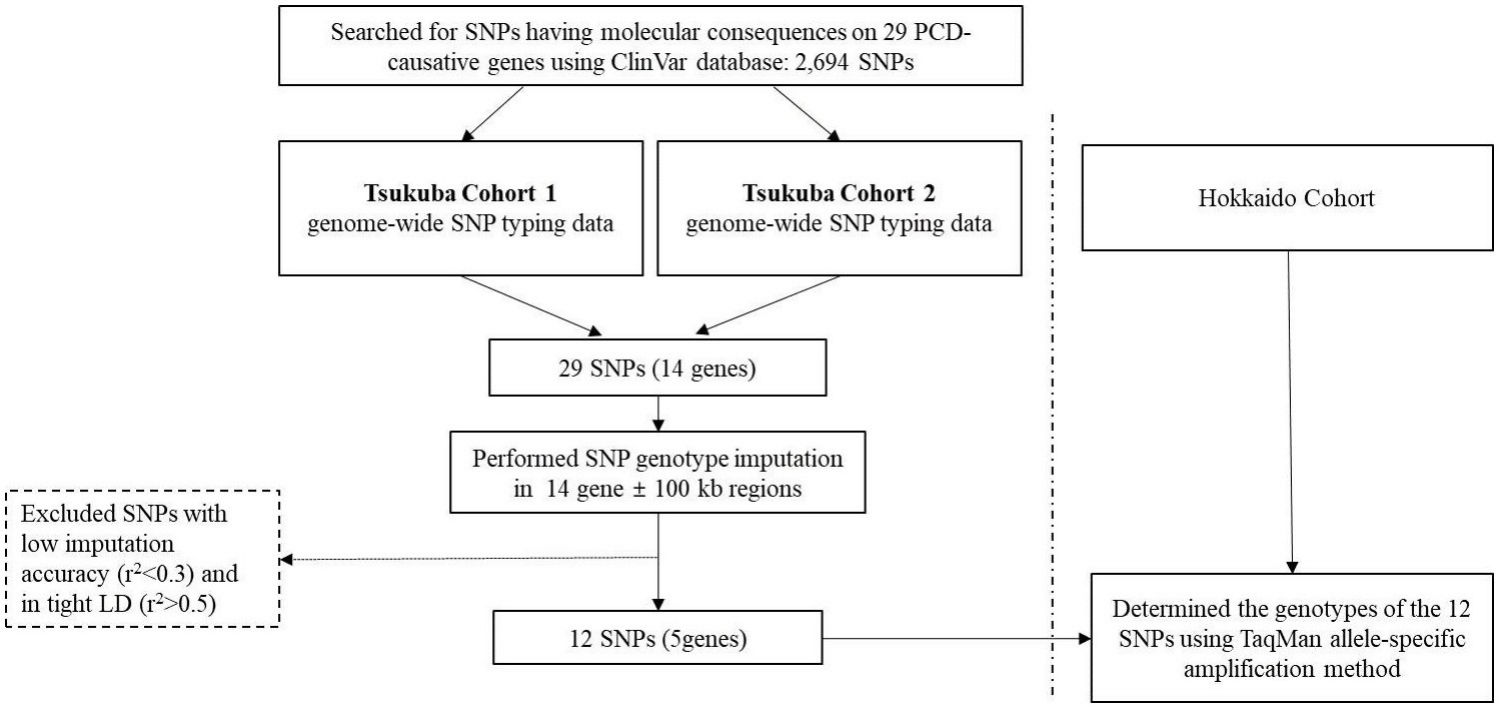
Flow diagram of the study design. PCD, primary ciliary dyskinesia; SNP, single nucleotide polymorphism; LD, linkage disequilibrium.

Although over 40 disease-causing genes have been described to date, this study focused on 29 functionally well-characterized causative genes of PCD (S5 Table) [6]. Using the freely accessible ClinVar database (https://www.ncbi.nlm.nih.gov/clinvar/) [27], a public archive of reports of the relationships among human variations and phenotypes with supporting evidence, we searched for SNPs that have been reported to cause molecular consequences in the 29 PCD-related genes, and we identified a total of 2,694 SNPs as of April 7, 2020. After exclusion of rare SNPs with minor allele frequencies (MAFs) < 0.1 in the Japanese population, SNPs were cross-referenced with our two sets of genome-wide genotyping SNP data (Tsukuba Cohort 1 and 2); a total of 29 SNPs at 14 genes remained. Since the genotyping platforms differed between Tsukuba Cohorts 1 and 2, genotype imputation in the 14 genes ± 100 kb regions of both cohorts was performed using the other cohort’s SNP data as the reference panel. We used a two-step imputation approach. First, imputation with pre-phasing of the target dataset was performed. The haplotypes for each individual were estimated using MACH software (pre-phasing). Then, the genotype imputation with pre-phased haplotypes in the MACH framework was performed by Minimac3 [22]. After excluding SNPs with low estimated imputation accuracies (Minimac r^2^ < 0.3) or SNPs in tight linkage disequilibrium (LD) (r^2^ > 0.5), 12 SNPs at 5 genes remained and were used for the further analysis (Fig 2, S2 Table).

### Calculating the multi-SNP genetic risk score (GRS)

We calculated the GRS of PCD-related genes using nonsense and missense mutations that cause protein loss or structural changes and mutations in the 3’-untranslated region (UTR), which is involved in mRNA stability and translation efficiency. Since pathogenic mutations of PCD are extremely rare, we focused on common SNPs that have not been reported to be involved in PCD pathogenesis, but may cause some functional effects on PCD-related genes. We then used PCD-GRS as a stratifying factor for cluster analysis.

As a measure of the cumulative effect of genes responsible for ciliary function, we calculated the PCD-GRS as a sum of the number of alleles causing molecular consequences at 12 loci for all individuals. Individual PCD-GRS values were calculated using the formula below:

$$PCD‐GRSi=\sum_{k=1}^{12}RAk$$

where PCD-GRS*i* is the GRS for individual *I*, and RA*k* is the number of risk alleles at SNP*k*. The PCD-GRS showed a normal distribution (S1 Fig).

### Statistical analysis

To identify asthma phenotypes influenced by PCD-related genes, we conducted a two-step cluster analysis of patients with asthma using 3 variables (PCD-GRS, forced expiratory volume in 1 second (%predicted FEV_1_), and age of onset) and IBM SPSS Statistics 26. According to our previous cluster analysis of adult asthma in the Japanese population, age at onset and %predicted FEV_1_ were the most important discriminators for asthma [28]. Indeed, several other cluster analyses have also identified 3 to 5 asthma phenotypes, with similarities among asthma phenotypes according to age of onset and lung function [29, 30]. Therefore, in the present study, we intentionally selected these variables in addition to the PCD-GRS. The similarity between each cluster was measured using log likelihood, and the optimal number of clusters was defined using Bayesian Information Criterion (BIC) values (https://www.ibm.com/docs/en/spss-statistics/24.0.0?topic=base-twostep-cluster-analysis). PCD-GRS values of identified clusters and healthy participants in each population were compared by two-tailed one-way analysis of variance (ANOVA) followed by Tukey’s post hoc analysis.

To clarify the genetic influence of each gene or SNP on the asthma clusters with high PCD-GRS and low PCD-GRS, we conducted a GWAS in the Tsukuba Cohorts 1 and 2, followed by meta-analysis. Quality control of genotyping data was conducted under the condition of MAF ≥ 1%, Hardy-Weinberg equilibrium test p ≥ 1.0 x 10^−6^, sample call rate > 90%, and genotyping call rate > 90% using PLINK1.90 [31]. The numbers of SNPs were as follows: Tsukuba Cohort 1, 466,404; Tsukuba Cohort 2, 480,188; and meta-analysis, 66,993. Since *DNAH5* was the gene with the strongest statistically significant association on meta-analysis, we further searched for SNPs with a significant difference in allele frequencies using SNP data within the *DNAH5* ±100 kb region, where SNP density was increased by imputation.

We used a generalized linear model to examine the gene-gene interactions between *DNAH5* rs1530496 and the other 8 SNPs on different chromosomes (rs12623642, rs3795958, rs2285943, rs10224537, rs2214326, rs7971, rs10250905, and rs600753) that were used to calculate the PCD-GRS.

## Supporting information

S1 Table Characteristics of healthy participants and patients with asthma in this study. (DOCX)

S2 Table Associations between the 12 SNPs and the prevalence of asthma (adjusted for sex, age, and smoking index). (DOCX)

S3 Table Characteristics of combined asthma clusters with high, low, and average PCD-GRS values in 673 patients who had never smoked. (DOCX)

S4 Table Clinical features related to mucociliary dysfunction according to the PCD-GRS. (DOCX)

S5 Table Twenty-nine PCD-related genes. (DOCX)

S1 Fig Histogram and normal Q-Q plot of the PCD-GRS in healthy participants (N = 2203, mean 12.54). *PCD-GRS*, primary ciliary dyskinesia-genetic risk score.
(DOCX)

S1 Data Minimal dataset. (XLSX)

## Abbreviations

DPB: diffuse panbronchiolitis
eQTL: expression quantitative trait locus
FeNO: fractional exhaled nitric oxide
FEV_1_: forced expiratory volume in 1 second
GRS: genetic risk score
GWAS: genome-wide association study
MAF: minor allele frequency
ODA: outer dynein arm
PCD: primary ciliary dyskinesia
SNP: single nucleotide polymorphism
UTR: untranslated region mutation
