# Supplementary material for: The primary ciliary dyskinesia-related genetic risk score is associated with susceptibility to adult-onset asthma
Source: PLoS One. 2024 Mar 8;19(3):e0300000. doi: 10.1371/journal.pone.0300000 (PMC10923447; doi:10.1371/journal.pone.0300000)
Supplement: S1 Table — (DOCX) [file pone.0300000.s001.docx]

**Supplementary Table 1.** Characteristics of healthy participants and patients with asthma in this study.

|  | **Tsukuba Cohort 1** | | **Tsukuba Cohort 2** | | **Hokkaido Cohort** | |
| --- | --- | --- | --- | --- | --- | --- |
|  | Healthy  participants  (n = 565) | Patients  with asthma  (n = 475) | Healthy  participants  (n = 965) | Patients  with asthma  (n = 237) | Healthy  participants  (n = 673) | Patients  with asthma  (n = 446) |
| PCD-GRS - mean (SD) | 12.71 (2.1) | 12.57 (2.13) | 12.50 (2.08) | 12.60 (2.05) | 12.46 (2.15) | 12.37 (2.09) |
| Age - years, median (range) | 53 (22-78) | 65 (19-90) | 51 (27-74) | 56 (20-75) | 48 (11-79) | 56 (16-84) |
| Female - n (%) | 271 (48) | 296 (55.1) | 526 (54.5) | 143 (59.1) | 312 (46.4) | 263 (59) |
| Age of onset - years, median (range) | - | 47 (1-87) | - | 41 (0-70) | - | 39 (0-77) |
| BMI - mean (SD) | 22.97 (3.3) | 23.64 (4.05) | 23.09 (3.00) | 23.43 (3.96) | - | - |
| %predicted FEV_1_ - mean (SD) | 90.04 (13.72) | 81.59 (24.1) | 93.31 (12.05) | 89.91 (19.97) | 105.03 (16.52) | 80.91 (22.48) |
| Z - score FEV_1_ - mean (SD) | -0.83 (1.39) | -1.64 (1.75) | -0.38 (0.98) | -1.09 (1.56) | -0.06 (1.31) | -2.05 (1.88) |
| FEV_1_/FVC - mean (SD) | 81.27 (6.63) | 69.26 (13.16) | 83.22 (5.19) | 74.9 (11.06) | 82.67 (7.26) | 68.36 (13.14) |
| Smoking status - n (%) |  |  |  |  |  |  |
| Never | 252 (44.6) | 245 (52.2) | 607 (62.9) | 194 (81.9) | 346 (56.3) | 235 (52.9) |
| Ex- | 202 (35.8) | 186 (39.7) | 197 (20.4) | 14 (5.9) | 94 (15.3) | 133 (30) |
| Current | 111 (19.6) | 38 (8.1) | 161 (16.7) | 29 (12.2) | 175 (28.5) | 76 (17.1) |
| Smoking index* – n (%) |  |  |  |  |  |  |
| 1-200 | 199 (35.3) | 72 (15.3) | 126 (13.1) | 40 (16.9) | 44 (7.5) | 64 (15.8) |
| >200 | 112 (19.9) | 155 (32.8) | 232 (24) | 3 (1.3) | 194 (33.2) | 106 (26.2) |
| Atopy† - n (%) | 252 (61.6) | 205 (72.2) | 540 (56) | 144 (71.3) | 354 (52.8) | 244 (55.3) |
| Total serum IgE (log) - mean (SD) | 1.90 (0.64) | 2.23 (0.65) | 1.73 (0.56) | 2.22 (0.61) | 1.83 (0.68) | 2.27 (0.68) |
| Eosinophilic asthma‡ - n (%) | - | 149 (42.7) | - | 102 (46.4) | - | 246 (58.7) |

Because information on age of onset and % predicted FEV_1_, which are variables used for cluster analysis, was missing for 62 and 5 patients with asthma in Tsukuba Cohort 1 and 2, we excluded these patients from the table. *Smoking index was calculated for current and past smokers by multiplying smoking dose (cigarettes per day) and duration (years smoked). †Atopy was defined as a positive response (>1.0 lumicount) to at least one of the 14 inhaled allergens. ‡Eosinophilic asthma is defined as a peripheral blood eosinophil count of more than 300 /μL or 5%. In patients with asthma in Tsukuba Cohort 1, information on smoking status, presence of atopy, total serum IgE, presence of allergic rhinitis, and presence of eosinophilic asthma was missing for 6, 53, 30,125, and 126 individuals, respectively. In healthy participants in Tsukuba Cohort1, information on FEV_1_/FVC and presence of atopy was missing for 5 and 156 individuals, respectively. In patients with asthma in Tsukuba Cohort 2, information on presence of atopy, total serum IgE, presence of allergic rhinitis, and presence of eosinophilic asthma was missing for 27, 20, 16, and 17 individuals, respectively. In patients with asthma in Hokkaido Cohort, information on FEV_1_/FVC, total serum IgE, smoking status, presence of atopy, and presence of eosinophilic asthma was missing for 166, 42, 2, 5, and 27 individuals, respectively. In healthy participants in Hokkaido Cohort, information on %predicted FEV_1_, FEV_1_/FVC, total serum IgE, smoking status, and presence of atopy was missing for 180, 64, 4, 58, and 3 individuals, respectively. *BMI*, body mass index; *FEV_1_*, forced expiratory volume in 1 second; *FVC*, forced vital capacity; *GRS*, genetic risk score
