# Supplementary material for: The primary ciliary dyskinesia-related genetic risk score is associated with susceptibility to adult-onset asthma
Source: PLoS One. 2024 Mar 8;19(3):e0300000. doi: 10.1371/journal.pone.0300000 (PMC10923447; doi:10.1371/journal.pone.0300000)
Supplement: S2 Table — (DOCX) [file pone.0300000.s002.docx]

**Supplementary Table 2.** Associations between the 12 SNPs and the prevalence of asthma (adjusted for sex, age, and smoking index).

| Gene | Chr | dbSNP | ClinVar database | | | Tsukuba Cohort 1  (n = 1102) | | | | Tsukuba Cohort 2  (n = 1207) | | | | Hokkaido Cohort  (n = 859) | | | |
| --- | --- | --- | --- | --- | --- | --- | --- | --- | --- | --- | --- | --- | --- | --- | --- | --- | --- |
|  |  |  | Mutation | Amino acid change | Molecular consequence | A1 | MAF | OR | P value | A1 | MAF | OR | P value | A1 | MAF | OR | P value |
| *CCDC164* | 2 | rs12623642 | c.1897G>T | p.Val633Phe | missense | G | 0.18 | 0.88 | 0.28 | G | 0.16 | 0.90 | 0.43 | G | 0.16 | 1.1 | 0.40 |
| *CCDC164* | 2 | rs3795958 | c.1069A>G | p.Lys357Glu | missense | G | 0.33 | 0.99 | 0.93 | G | 0.37 | 0.91 | 0.36 | G | 0.37 | 1.2 | 0.19 |
| *DNAH5* | 5 | rs2277046 | c.12658A>G | p.Thr4220Ala | missense | C | 0.49 | 1.03 | 0.70 | C | 0.48 | 1.1 | 0.35 | G | 0.46 | 1.1 | 0.39 |
| *DNAH5* | 5 | rs10513155 | c.8586G>T | p.Leu2862Phe | missense | A | 0.18 | 1.2 | 0.18 | A | 0.14 | 0.91 | 0.53 | T | 0.15 | 1.05 | 0.73 |
| *DNAH5* | 5 | rs1530498 | c.1672A>G | p.Thr558Ala | missense | A | 0.23 | 1.2 | 0.16 | A | 0.24 | 0.98 | 0.86 | A | 0.22 | 0.91 | 0.47 |
| *DNAH5* | 5 | rs1530496 | c.71G>A | p.Gly24Glu | missense | G | 0.32 | 1.2 | 0.043 | G | 0.32 | 0.97 | 0.77 | G | 0.32 | 1.0 | 0.98 |
| *DNAH11* | 7 | rs2285943 | c.100G>T | p.Glu34Ter | nonsense | T | 0.35 | 1.1 | 0.35 | T | 0.35 | 1.05 | 0.66 | T | 0.27 | 1.1 | 0.36 |
| *DNAH11* | 7 | rs10224537 | c.3112A>G | p.Thr1038Ala | missense | A | 0.22 | 1.1 | 0.59 | A | 0.23 | 1.1 | 0.37 | A | 0.21 | 1.08 | 0.55 |
| *DNAH11* | 7 | rs2214326 | c.10399G>A | p.Ala3467Thr | missense | G | 0.17 | 1.04 | 0.74 | G | 0.17 | 0.98 | 0.89 | G | 0.16 | 0.84 | 0.21 |
| *DNAH11* | 7 | rs7971 | c.*88A>G | - | 3’-UTR | G | 0.18 | 1.01 | 0.92 | G | 0.18 | 1.1 | 0.41 | G | 0.18 | 0.88 | 0.33 |
| *TXNDC3* | 7 | rs10250905 | c.622T>C | p.Cys208Arg | missense | T | 0.42 | 1.0 | 0.96 | T | 0.44 | 1.0 | 0.99 | T | 0.43 | 1.1 | 0.31 |
| *DYX1C1* | 15 | rs600753 | c.572A>G | p.Glu191Gly | missense | T | 0.36 | 1.06 | 0.54 | T | 0.28 | 0.93 | 0.50 | T | 0.28 | 1.04 | 0.74 |

*Chr*, chromosome; *SNP*, single nucleotide polymorphism; *UTR*, untranslated region; *A1*, minor allele; *MAF*, minor allele frequency; *OR*, odds ratio.
