# Supplementary material for: The primary ciliary dyskinesia-related genetic risk score is associated with susceptibility to adult-onset asthma
Source: PLoS One. 2024 Mar 8;19(3):e0300000. doi: 10.1371/journal.pone.0300000 (PMC10923447; doi:10.1371/journal.pone.0300000)
Supplement: S3 Table — (DOCX) [file pone.0300000.s003.docx]

**Supplementary Table 3. Characteristics of combined asthma clusters with high, low, and average PCD-GRS values in 673 patients who had never smoked**

|  | Group1:  PCD-GRS-Hi asthma  (Combined group of T1-A, T2-A, -B, H-A) | Group2:  PCD-GRS-Lo asthma  (Combined group of T1-D, T2-D, -E, H-D, -E) | Group3:  PCD-GRS-average asthma  (Combined group of the other clusters) | P value‡ |
| --- | --- | --- | --- | --- |
| N | 202 | 217 | 254 |  |
| PCD-GRS - mean (SD) | 14.38 (1.29) | 11.07 (1.51) | 12.42 (1.88) | <0.001 |
| Female - n (%) | 153 (75.7) | 181 (83.4) | 166 (65.1) | <0.001 |
| Adjusted residual | 0.6 | 3.8 | -4.2 |  |
| Age - years, median (range) | 61 (27-84) | 62 (20-88) | 47 (16-84) | <0.001 |
| Age of onset - years, median (range) | 52.5 (19-82) | 50 (1-78) | 16 (0-78) | <0.001 |
| FEV_1_ %predicted - mean (SD) | 95.96 (19.0) | 92.89 (18.86) | 71.07 (20.70) | <0.001 |
| Z - score FEV_1_ - mean (SD) | -0.79 (1.47) | -1.08 (1.48) | -2.54 (1.86) | <0.001 |
| FEV_1_/FVC - mean (SD) | 74.78 (10.73) | 74.55 (10.47) | 68.37 (13.71) | <0.001 |
| Atopy* - n (%) | 112 (62.2) | 108 (54.0) | 191 (78.0) | <0.001 |
| Adjusted residual | -1.2 | -4.3 | 5.2 |  |
| Total serum IgE (log) - mean (SD) | 2.11 (0.58) | 2.0 (0.64) | 2.31 (0.63) | <0.001 |
| Eosinophilic asthma†- n (%) | 77 (45.0) | 102 (51.8) | 115 (56.9) | 0.072 |

PCD-GRS-Hi: combined clusters with higher PCD-GRS; PCD-GRS-Lo: combined clusters with lower PCD-GRS. *Atopy was defined as a positive response (>1.0 lumicount) to at least one of the 14 inhaled allergens. †Eosinophilic asthma was defined as a peripheral blood eosinophil count of more than 300 /μL or more than 5%. ‡For PCD-GRS, difference was found between all groups. For age, onset age, %predicted FEV_1_, FEV_1_/FVC, and total serum IgE, difference was found between GRS-Hi group and the -average group, and between GRS-Lo group and the -average group. For categorical covariates, adjusted residuals are shown in the table. *FEV_1_*, forced expiratory volume in 1 second; *FVC*, forced vital capacity; *PCD*, primary ciliary dyskinesia; *GRS*, genetic risk score.
