# Supplementary material for: The primary ciliary dyskinesia-related genetic risk score is associated with susceptibility to adult-onset asthma
Source: PLoS One. 2024 Mar 8;19(3):e0300000. doi: 10.1371/journal.pone.0300000 (PMC10923447; doi:10.1371/journal.pone.0300000)
Supplement: S4 Table — (DOCX) [file pone.0300000.s004.docx]

**Supplementary Table 4. Clinical features related to mucociliary dysfunction according to the PCD-GRS**

|  | **Group1:**  **PCD-GRS-Hi asthma**  (Combined group of T1-A, 　　T2-A, -B, H-A) | **Group2:**  **PCD-GRS-Lo asthma**  (Combined group of T1-D, 　　T2-D, -E, H-D, -E) | **Group3:**  **PCD-GRS-average asthma**  **(**Combined group of the other clusters**)** | P value |
| --- | --- | --- | --- | --- |
| Antibiotic use due to respiratory infection in past 12 months –n (%) | 16 (14.7) | 12 (12.0) | 29 (18.1) | 0.41 |
| Previous long-term use of low-dose macrolide therapy –n (%) | 4 (3.7) | 4 (4.0) | 15 (9.4) | 0.12 |
| Blood neutrophil count (%) –median (quantile) | 60.31 (53-66.4) | 60.0 (52.9-68.2) | 62.9 (55-69.7) | 0.16 |

PCD-GRS-Hi: combined clusters with higher PCD-GRS; PCD-GRS-Lo: combined clusters with lower PCD-GRS. Information on previous antibiotic use due to respiratory infection in past 12 months and low-dose macrolide therapy was available in 369 patients (109, 100 and 160 in Group 1, 2 and 3, respectively), and information on blood neutrophil count (%) was available in 425 patients (137, 118 and 170 in Group 1, 2 and 3, respectively)*. PCD*, primary ciliary dyskinesia; *GRS*, genetic risk score.
