# Supplementary material for: The primary ciliary dyskinesia-related genetic risk score is associated with susceptibility to adult-onset asthma
Source: PLoS One. 2024 Mar 8;19(3):e0300000. doi: 10.1371/journal.pone.0300000 (PMC10923447; doi:10.1371/journal.pone.0300000)
Supplement: S5 Table — (DOCX) [file pone.0300000.s005.docx]

**Supplementary Table 5.** Twenty-nine PCD-related genes.

| **Gene name** | **Chromosome** | **Position (bp), hg19** |
| --- | --- | --- |
| Outer dynein arm truncation | | |
| *DNAH5* | 5 | 13,690,437-13,944,589 |
| *TXNDC3/NME8* | 7 | 37,888,199-37,940,002 |
| *DNAI1* | 9 | 34,458,811-34,520,982 |
| *DNAI2* | 17 | 72,270,386-72,311,023 |
| *DNAL1* | 14 | 74,111,578-74,170,431 |
| *CCDC114* | 19 | 48,799,709-48,823,332 |
| *ARMC4* | 10 | 28,101,097-28,287,977 |
| *CCDC151* | 19 | 11,531,272-11,545,980 |
| Outer dynein arm defect | | |
| *CCDC103* | 17 | 42,977,080-42,981,047 |
| Outer and inner dynein arm truncation | | |
| *LRRC6* | 8 | 133,584,201-133,687,863 |
| *HEATR2* | 7 | 766,338-826,116 |
| *DYX1C1* | 15 | 55,722,506-55,800,432 |
| *DNAAF1* | 16 | 84,178,865-84,211,524 |
| *DNAAF3* | 19 | 55,670,029-55,678,090 |
| *DNAAF2* | 14 | 50,091,892-50,101,948 |
| *SPAG1* | 8 | 101,170,263-101,254,132 |
| *C21orf59* | 21 | 33,973,984-33,984,918 |
| *ZMYND10* | 3 | 50,378,537-50,383,156 |
| Central apparatus defect | | |
| *HYDIN* | 16 | 70,841,287-71,264,625 |
| *RSPH4A* | 6 | 116,937,642-116,954,148 |
| Radial spoke defects | | |
| *RSPH9* | 6 | 43,612,767-43,638,748 |
| *RSPH1* | 21 | 43,892,597-43,916,401 |
| Nexin-dynein regulatory complex defect | | |
| *CCDC164/DRC1* | 2 | 26,624,784-26,679,579 |
| Variable axonemal disorganization | | |
| *CCDC39* | 3 | 180,331,796-180,397,283 |
| *CCDC40* | 17 | 78,010,431-78,074,412 |
| Rare cilia | | |
| *CCNO* | 5 | 54,526,981-54,529,508 |
| *MCIDAS* | 5 | 54,515,425-54,523,143 |
| Normal axonemal ultrastructure | | |
| *DNAH11* | 7 | 21,582,833-21,941,186 |
| *CCDC65* | 12 | 49,297,893-49,315,359 |

*PCD*, primary ciliary dyskinesia; *bp*, base pair
