# Supplementary material for: The primary ciliary dyskinesia-related genetic risk score is associated with susceptibility to adult-onset asthma
Source: PLoS One. 2024 Mar 8;19(3):e0300000. doi: 10.1371/journal.pone.0300000 (PMC10923447; doi:10.1371/journal.pone.0300000)
Supplement: S1 Fig — PCD-GRS, primary ciliary dyskinesia-genetic risk score. (DOCX) [file pone.0300000.s006.docx]

**
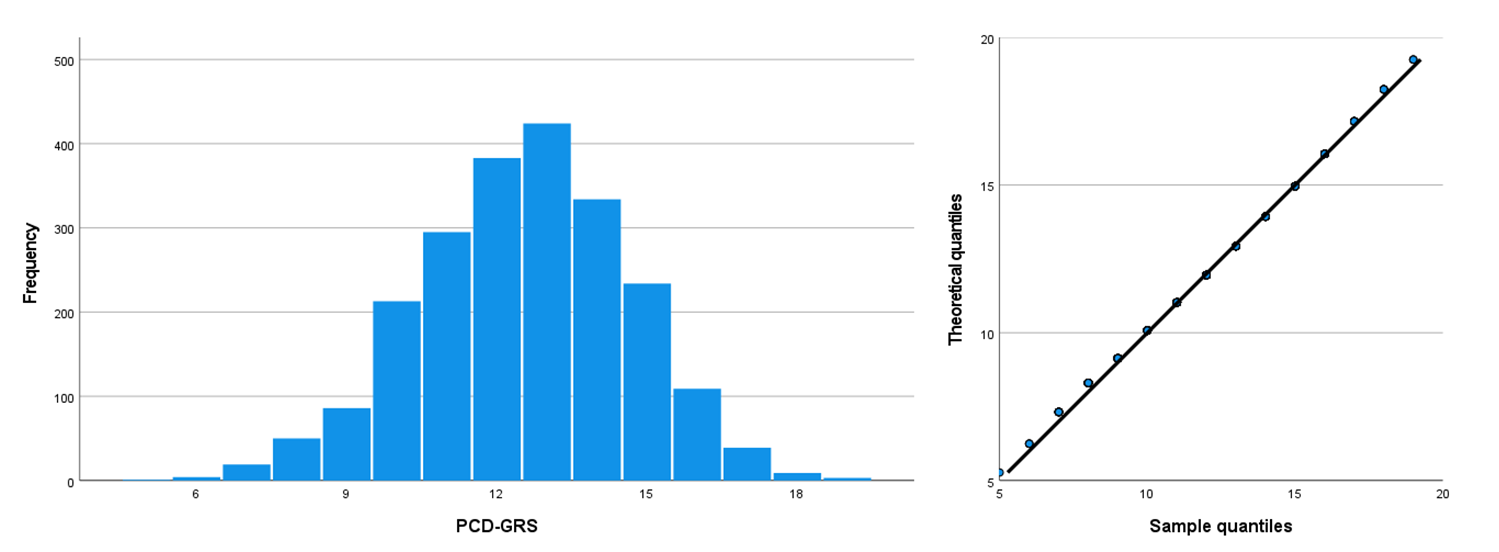
**

**Supplementary Figure 1.** Histogram and normal Q-Q plot of the PCD-GRS in healthy participants (N = 2203, mean 12.54). *PCD-GRS*, primary ciliary dyskinesia-genetic risk score.
